# Crystallization characteristics in supercooled liquid zinc during isothermal relaxation: A molecular dynamics simulation study

**DOI:** 10.1038/srep31653

**Published:** 2016-08-16

**Authors:** Li-li Zhou, Rang-su Liu, Ze-an Tian, Hai-rong Liu, Zhao-yang Hou, Ping Peng

**Affiliations:** 1 School of Physics and Microelectronics Science, Hunan University, Changsha, 410082, China; 2 Department of Information Engineering, Gannan Medical University, Ganzhou, 341000, China; 3 College of Materials Science and Engineering, Hunan University, Changsha, 410082, China; 4 Department of Applied Physics, Chang’an University, Xi’an 710064, China

## Abstract

The crystallization characteristics in supercooled liquid Zn during isothermal relaxation were investigated using molecular dynamics simulations by adopting the cluster-type index method (CTIM) and the tracing method. Results showed that the crystallization process undergo three different stages. The size of the critical nucleus was found to be approximately 90–150 atoms in this system; the growth of nuclei proceeded via the successive formation of hcp and fcc structures with a layered distribution; and finally, the system evolved into a much larger crystal with a distinct layered distribution of hcp and fcc structures with an 8R stacking sequence of ABCBACAB by adjusting all of the atoms in the larger clusters according to a certain rule.

The crystallization process of liquid metals has attracted significant attention for many years due to its importance in the materials processing field and due to interest in certain predictable properties^1^. Specifically, researchers are interested in understanding the crystallization characteristics and controlling the formation of the resulting structures^2^,^3^,^4^. Therefore, many works^5^,^6^,^7^,^8^,^9^,^10^,^11^,^12^,^13^,^14^,^15^,^16^ have paid attention to the structures of the nuclei formed during the crystallization process, and a variety of results have been obtained. Scope and Andersen^5^ found that critical nuclei can contain both fcc and hcp particles in a supercooled Lennard-Jones liquid. Ten Wolde *et al*.^6^ observed the formation of a pre-critical nucleus with a bcc structure during the crystallization process, and as the nucleus grew to its critical size, it developed a stronger fcc order in its core, whereas its surfaces still retained a bcc structure. A simulation study^7^ on supercooled liquid Ni_6_Cu_4_ melt indicated that the nucleus was a random mixture of fcc and hcp structures. Recently, the nucleation of a polymorph was directly observed in experiments^8^,^9^; later, this finding was also demonstrated by computer simulations of colloidal suspensions^10^ and a Lennard-Jones system^11^. A simulation study on the Lennard-Jones system observed that, before nucleation, thermal fluctuations prefer hcp-type atomic arrangements over fcc-type, whereas during the nucleation and growth stages, this preference is reversed^13^. Several nucleus-forming mechanisms have been proposed to account for this phenomenon. Some researchers have attributed this phenomenon to the competing processes of homogenous nucleation^5^,^6^ among different types of crystal structures, while other researchers have ascribed the phenomenon to the heterogeneous nucleation (or cross-nucleation) of one type of crystal structure on another^8^,^9^,^10^,^11^,^12^. These studies of different systems have provided important information toward helping the field to understand the mechanisms of crystal nucleation and growth. However, a variety of open questions remain concerning the very details of the nucleation and growth processes in supercooled liquid metals.

The main purpose of this work was to reveal the crystallization characteristics, especially the nucleation and growth processes, and the corresponding microstructural evolution in supercooled liquid metal Zn. The growth of nuclei in supercooled liquid Zn was found to proceed through the successive formation of hcp and fcc atomic layers. Because this nucleation process is completed in a very short time, it is well known that investigating the details of this process poses a difficult experimental challenge. However, molecular dynamics (MD) simulations offer an effective remedy. In this work, the formation and evolution processes of the crystal nuclei in supercooled liquid Zn metal were studied using MD simulations and were analyzed using the cluster type index method (CTIM), the tracing method and 3D visual analysis. Therefore, the crystallization processes, especially the microstructural evolution of nuclei in supercooled liquid Zn, are clearly elucidated in detail in this work and several new findings are presented.

## Results

### The validation of the simulation

**Figure 1:**
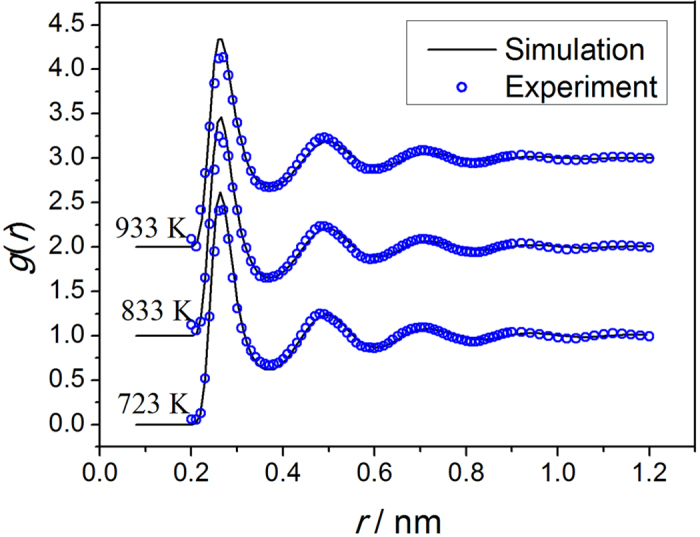
Comparison of the simulation *g*(*r*) curves (solid lines) with the experimental data (circles)^17^ for Zn at different temperatures.

The pair distribution function *g*(*r*) can be obtained through a Fourier transformation of the X-ray diffraction factor *S*(*q*), so the validity of the current simulation can be verified by comparing the calculated *g*(*r*) curves with the experimental results obtained by Waseda^17^, as shown in Fig. 1. The *g*(*r*) was calculated under the constant pressure of P = 1 atm, and the density was adjusted to maintain the required pressure. As it can be seen, the calculated results are in good agreement with the experimental results, demonstrating that the effective pair potential function adopted here is a successful approach for describing the objective physical nature of the microstructure of this Zn system.

### Three stages of crystallization

**Figure 2:**
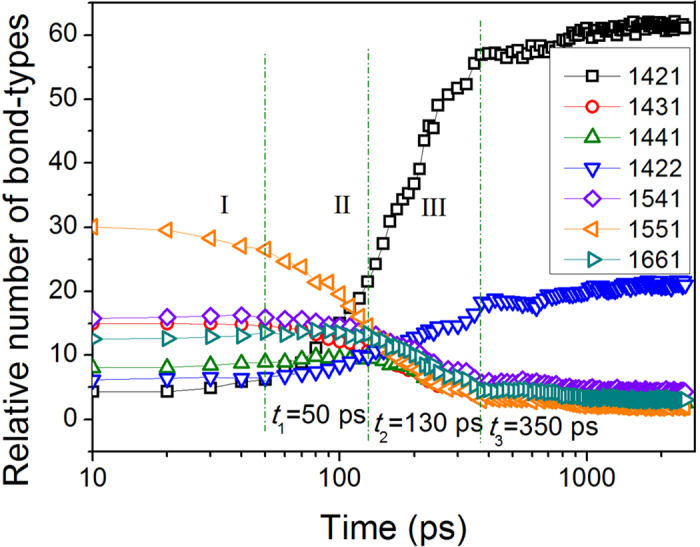
The relative number of bond types as a function of relaxation time at 353 K, with the three important time points labeled using dashed lines.

The details of the microstructural evolution during the relaxation process of the supercooled liquid can be obtained using a HA bond-type analysis^18^, as described in refs 19, 20, 21. The changes in the relative numbers of the main bond-types with the relaxation time are shown in Fig. 2. It can be clearly seen that the system starts from a disordered structure with significant fractions of 1551, 1541, and 1431 bond-types and almost remains constant over the course of the initial 50 ps. With an increase in the relaxation time, the proportion of the 1421 bond type increases rapidly, and the 1422 bond type concomitantly increases slowly. In contrast, the other bond-types, such as 1541, 1431, 1441 and 1661, decrease slightly, whereas the 1551 bond type exhibits a much greater decrease. These changes imply that the system is undergoing the crystallization process. At approximately 350 ps, all bond types in the system reach a plateau, indicating that the crystallization process is complete and that the system has crystallized into a coexisting structure dominated by the hcp and fcc atoms with 1421 and 1422 bond types.

**Figure 3:**
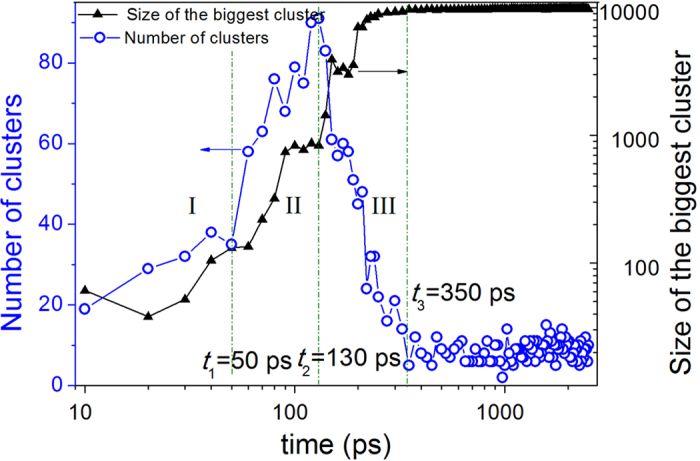
Relationship of the number of crystal clusters (open circles) and the maximum size of the cluster (solid triangles) with relaxation times at 353 K.

However, the HA bond-type index can only be used to describe small structural units consisting of fewer than 10 atoms and is incapable of analyzing the crystallization mechanism. To obtain further insight into the formation and evolution of nuclei in supercooled liquid Zn during isothermal relaxation, the cluster-type index method (CTIM-2)^21^,^22^ was adopted. Based on CTIM-2, the number of clusters and the size of the maximum cluster were obtained, as shown in Fig. 3. Over the course of the initial 50 ps, the number of crystal clusters increased slightly, and the size of the largest cluster remained very small, suggesting that several small embryos had formed in the supercooled liquid. These small embryos consisted of either a single or several basic clusters and contained approximately twenty atoms. Such clusters can form and disaggregate at random due to thermal fluctuations in the system. In the following time interval (*t*_1_ < *t* < *t*_2_), both the number of clusters and the size of the biggest crystal cluster increased significantly, which means that small nuclei had grown into medium-size nuclei in the supercooled liquid. In the time interval *t*_2_ < *t* < *t*_3_, the number of crystal clusters decreased continually, and the size of the largest cluster increased to an overwhelming degree, implying that the medium-size crystal clusters had amalgamated into even bigger clusters; that is, the prominent growth of the biggest cluster was mainly attributed to the combination of several crystal clusters and the growth of the existing nuclei. Consequently, a large crystal cluster containing 97.7% of the total 10,000 Zn atoms had finally formed in the system at this point. After *t*_3_, the relative number of bond types, as shown in Fig. 2, and the size of the largest cluster and the number of clusters, as shown in Fig. 3, reached a plateau, indicating that the process of crystallization was complete.

From the analysis above, the crystallization process of supercooled liquid Zn, as demonstrated in our previous work^23^ on the crystallization process of amorphous metal Na, can be divided into three stages by increasing the relaxation time: stage I (0 < *t* < 50 ps) corresponds to the formation process of the critical nucleus from the supercooled liquid; stage II (50 ps < *t* < 130 ps) corresponds to the process of nucleus growth; and stage III (130 ps < *t* < 350 ps) corresponds to a the process of grain coarsening due to the growth of the existing nuclei and nuclei merging with other crystal grains.

### Formation and evolution of nuclei

**Figure 4:**
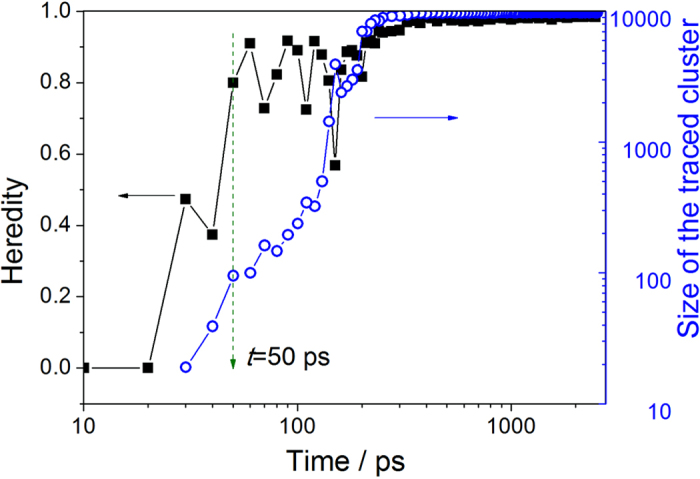
Relations of the heredity and the size of the traced cluster with the relaxation times.

The tracing method was used to develop a deeper understanding of the mechanisms of formation and evolution of nuclei in the system, and the relations of the heredity and the size of the traced cluster with the relaxation time were obtained, as shown in Fig. 4, which clearly shows the crystallization pathway of the supercooled liquid. During the initial 20 ps, very few crystal clusters are present in the supercooled liquid, and these clusters are unstable and cannot be inherited, so both the heredity and the size of the traced cluster remain at zero. In the next increment of 20 ps, the heredity of the traced cluster remains below 50%, suggesting that the cluster is still unstable and that the system is undergoing violent atomic movements and structural rearrangement. At *t* = 50 ps, the size of the traced cluster reaches 95 atoms, and the proportion of the inherited atoms within the cluster remains greater than 50%, indicating that a steady increase has been attained.

This suggests that the traced cluster begins to grow stably and can be regarded as a critical nucleus. It can be clearly seen that in this system, the size of the critical nucleus is approximately 90–150 atoms. The fluctuations of heredity within stage I and stage II indicate that the nucleation and growth process is kinetic: some atoms coalesce into the nucleus, while others disjoin from it.

**Figure 5:**
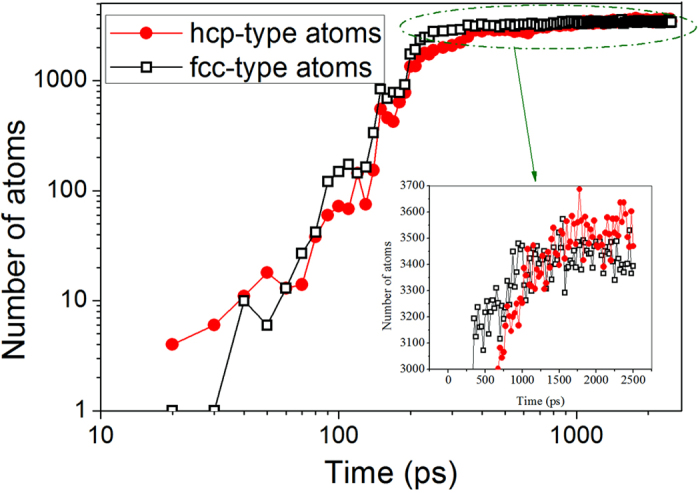
Structure evolution of the traced cluster (nucleus).

Moreover, the internal structure of the traced cluster was analyzed using CTIM-2, as shown in Fig. 5. With an increase in the relaxation time, both the hcp- and fcc-type atoms in the traced nucleus increased, indicating that the nucleus in the system is mainly composed of hcp and fcc atoms. This phenomenon has also been found in the nucleation process of Ni_6_Cu_4_ 7 alloy, crystalline N_2_ 12, and hard-sphere systems^14^ in which the nuclei are also mainly formed on the basis of hcp and fcc structures. However, these results are different from those obtained by Wolde^6^, who studied a Lennard-Jones system in which the precritical nuclei were mainly formed with bcc-ordered structures and become more fcc-ordered in the core as the nuclei grew to their critical size. According to our simulation, no bcc structures appeared throughout the entire nucleation process.

Figure 5 clearly shows that the growth of the nucleus proceeds through a successive increase in hcp and fcc atoms. The number of fcc atoms (*N*_fcc_) increases concomitantly with that of hcp (*N*_hcp_) during nucleation and growth. *N*_fcc_ grows slightly faster than *N*_hcp_. With the increase of the relaxation time, *N*_hcp_ still grows sluggishly, whereas *N*_fcc_ remains a constant value, as shown in the insert of Fig. 5. This is not surprising because hcp is the stable phase for Zn. Finally, *N*_hcp_ exceeds *N*_fcc_ at approximately 1575 ps. The early stage of crystallization is shown to be mainly controlled by kinetics (e.g., the relative growth rate of crystals), but in the final structure of the crystal, the relative stability of the crystal phase plays a key role; specifically, the system is assumed to transform into the most stable hcp phase if the relaxation time is sufficiently long. This observation is consistent with Oxtoby’s opinion^24^ that a critical nucleus does not need to have the same internal structure as that of the stable bulk phase that eventually forms. Because the fcc and hcp structures have an almost equivalent free energy, they are likely to appear in the nucleus successively^11^,^12^,^25^, whereas if given a sufficiently long relaxation time, a more thermodynamically stable phase should grow faster and become dominant in the final solidified structure.

**Figure 6:**
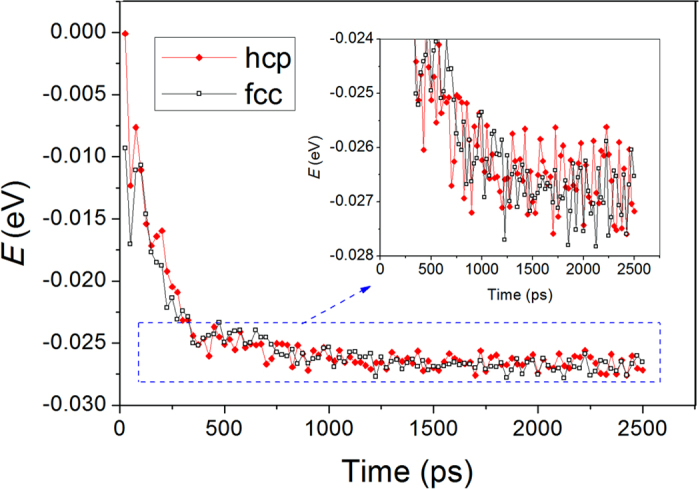
Average energy per atom of the hcp and fcc atoms in the system; the insert is an enlargement of the figure in the blue dashed rectangle.

Our results also indicate that the average energy per hcp or fcc atom fluctuates at almost the same level, i.e., they have little difference in this aspect, as shown in Fig. 6. In the initial relaxation period, both hcp and fcc basic clusters in the supercooled liquid Zn are loose and defective, so their average energy is high. As the hcp and fcc structures become more and more perfectly crystalline, the average energy per atom decreases with the increase in the relaxation time.

**Figure 7:**
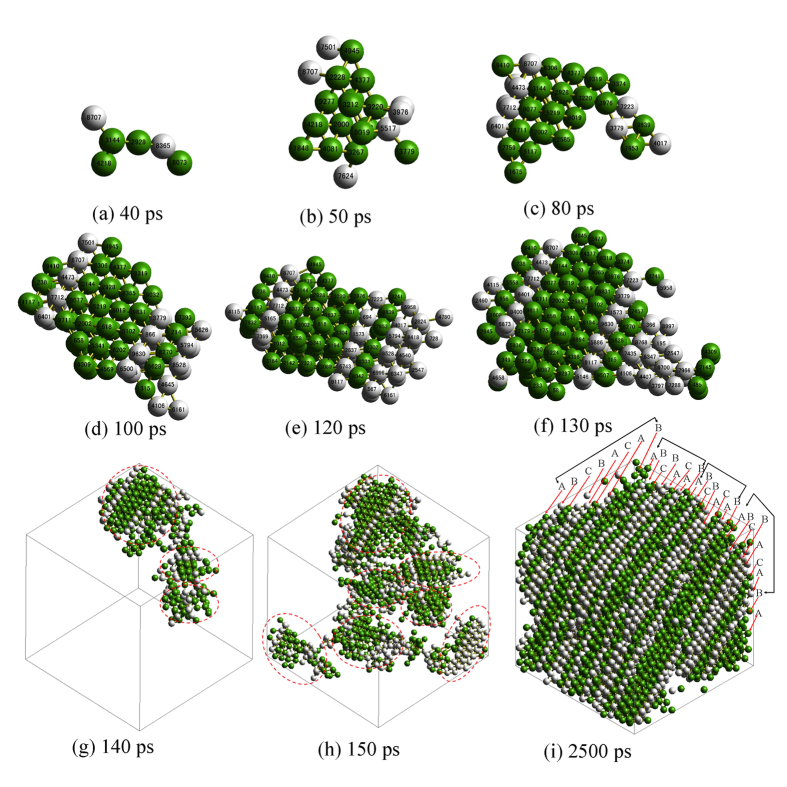
Snapshots of the evolution of the traced cluster at different relaxation times. [Only the central atoms of a basic cluster are shown, where green indicates hcp atoms, gray indicates fcc atoms, and the red dashed ellipses in (**g**,**h**) mark the different nuclei that are the constituents of the large nucleus].

As mentioned above, the hcp and fcc structures coexist in the traced nucleus. However, the overarching arrangement of the different structural types is not yet known. Fortunately, with the help of 3D graphics techniques, the details of the structural evolution can be presented intuitively, as shown in Fig. 7. The nucleation and growth processes occur through the successive formation of the layered distribution of hcp and fcc structures. This is different from the results of Leyssale and O’Malley *et al*.^12^,^14^, who indicate that the hcp and fcc atoms in the nucleus have a great tendency for block-like distribution; this finding is also different from the case of a random mixture of two types of structures^7^. These differences in the arrangement of nuclei might be induced either by different crystallization conditions or by the essential distinctness of different particle systems, or by a combination of the two.

From 3D visual analysis, the formation and growth of the nucleus can be observed more intuitively, further confirming that the crystallization process exhibits three stages. Stage I corresponds to the nucleation process in which the embryo is very small and consists of only several basic clusters indexed by CTIM-2, and the shape of the embryo is chainlike (Fig. 7(a)), such that the internal structure is loose and unstable. Stage II corresponds to the nuclei growth process in which the chainlike embryo becomes bigger and evolves into a more compact shape at approximately 50 ps (Fig. 7(b)) and then grows steadily with increasing time. This result is consistent with the tracing analysis result and further confirms that the critical nucleus is formed at approximately 50 ps. Moreover, it can be clearly seen in Fig. 7(b–f) that the nucleus grows by adding atoms to the core in a layer-like fashion, where the hcp and fcc atoms are added to their respective matrix core with the introduction of some stacking faults. Stage III corresponds to the coarsening process. As shown in Fig. 7(g–i), the nucleus continues to coarsen, and the individual nuclei begin to coalesce into each other. It can also be found from Fig. 7(g,h) that the growth orientations of different medium-size nuclei marked with red dashed ellipses vary relative to each other, namely, that the growing orientation of the traced nucleus, which consists of many medium-size nuclei, is localized, such that the distribution of hcp and fcc structures in the traced nucleus appears slightly disordered. With an increase in the relaxation time, the nucleus continues adjusting its arrangement and evolves into a more perfectly layered distribution. Here, we refer to the fcc layer as f and the hcp layer as h for short; accordingly, as shown in Fig. 7(i), from the top left corner to the bottom right corner, the stacking sequence is hfhffhfh hfhffhfh hfhffhfh hfhffhfh h.

**Figure 8:**
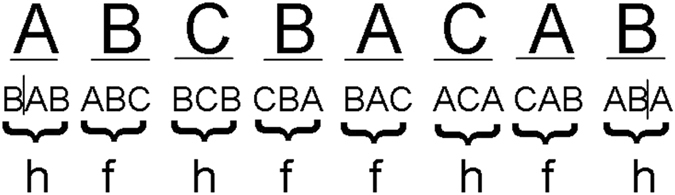
The stacking sequence of the 8R lattice in A-, B- and C-layer notation and h-f notation.

According to convention, the possible atom positions in the successive hexagonal layers are referred to as A, B and C; in this manner, the fcc lattice is described by the three-layer repeating sequence: ABCABC…, and the hcp lattice is described by the stacking sequence ABAB…^26^. Accordingly, the distribution of the layered structure can also be described by denoting each layer as h (hcp) or f (fcc) depending on its local environment^27^. For example, a B layer that has an A layer adjacent on one side and a C layer on the other side is denoted as an f layer; whereas a B layer that has an A layer adjacent to it on both sides is denoted as an h layer, as shown in Fig. 8.

Therefore, the layer stacking sequence of the zinc system in A-, B- and C-layer notation is ABCBACAB…. That is, a new 8R structure of the ABCBACAB… sequence with some stacking faults is finally formed in the Zn system, and this new structure is clearly different from the well-known 9R structure of the ABCBCACAB… sequence that was previously observed for Cu^28^ and Ag^29^. To our knowledge, few reports are available on the 8R crystal structure of metals; hence, our results provide an important suggestion of the possible crystallization structure of metals.

## Discussion

In this work, the crystallization characteristics during the process of relaxation of supercooled liquid Zn were investigated using molecular dynamics simulations and were analyzed using the cluster type index method (CTIM-2), the tracing method and 3D visual analysis. We briefly describe the main results obtained.

The whole crystallization process reveals three stages: stage I corresponds to the nucleation process in which small embryos are randomly formed in the supercooled liquid, and the critical nucleus appears at approximately 50 ps with a size of approximately 90–150 atoms; stage II corresponds to the growth process in which the nuclei continue growing, and meanwhile, new nuclei are also formed in the system; in stage III, the nuclei are continually coarsening due to the growth of existing nuclei and the merging of nuclei with each other, while the central atoms are bonded in different types of basic clusters to form larger crystal clusters.

Interestingly, we observed that the fcc and hcp structures grew concomitantly during the crystallization of liquid metal Zn despite the fact that Zn is an hcp crystal when in an equilibrium state. This phenomenon makes sense because the fcc and hcp phases are structurally compatible and have almost equivalent free energies. The coexistence of fcc and hcp structures with a single crystal has been widely observed in various systems^7^,^12^,^13^,^14^,^30^. However, the crystallization mechanism is different from that of some other systems in which the crystal nucleation first proceeds into the metastable bcc phase^6^,^22^. Such behavior was not observed for zinc.

Additionally, in our work, the fcc and hcp atoms in the nuclei are distributed in a layer-like fashion. The growth of the nuclei proceeds by adding atoms to the core layer-by-layer, while each of the hcp and fcc atoms is added to its respective matrix core with some stacking faults, which can be interpreted as follows: the free-energy difference between the fcc and hcp structures is very small^25^,^31^, and some studies^31^,^32^ have shown that the free-energy barrier of nucleation for both hcp and fcc structures is much less than that of either hcp or fcc structures, which can be overcome more easily by the structure fluctuation in the fluid.

By increasing the relaxation time, the system finally evolved into an 8R structure with the stacking sequence of ABCBACAB. To our knowledge, there have not yet been any reports of 8R structures in crystalline metals or alloys. Therefore, our simulation results suggest a possible novel crystallization structure for metals.

## Methods

### Simulation details

In this paper, the MD simulations were performed in a system consisting of 10,000 Zn atoms in a cubic box under periodic boundary conditions, developed using the *NVT* ensemble. Interactions among atoms were calculated using the effective pair potential function of the generalized nonlocal model-pesudopotential (GNMP) developed by Wang *et al*.^33^,^34^. The function is


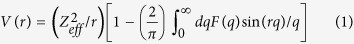


where Z_*eff*_ is the effective ionic valence and *F*(*q*) is the normalized energy wave number characteristic, both of which are defined in detail in refs 33,34. The pair potential was cut off at 20 a.u. (atomic units). The equations of motion were integrated using the leapfrog algorithm, and the time step was set to 5 fs.

The system was first melted and equilibrated at 973 K (the melting point of Zn is 692 K) by running 50,000 time steps and was then cooled down to the given temperature of 353 K at a cooling rate of 1 × 10^11^ K/s. In this simulation, the damped force method^35^,^36^ was adopted to control the system temperature. Finally, to investigate the early stage of crystallization during the isothermal relaxation process, the system was allowed to run another 500,000 time steps (=2500 ps) at the same temperature, and the configurations at given relaxation times (the time interval Δ*t* = 10 ps in the initial 250 ps and Δ*t* = 25 ps in the following relaxation time) were recorded for further analysis.

### HA bond-type index method

The HA bond-type index method was first developed by Honeycutt and Andersen^15^ to describe the microstructural characterization during the solidification process. In this method, four integers are used to describe the local configurations of two atoms. If the two atoms are bonded (their distance less than or equal to a certain bond-length, where here the bond-length is the distance to the first minimum in the pair distribution function), the first integer is 1; otherwise, it is 2. The second integer is the number of atoms shared by the two atoms. The third integer is equal to the number of bonds among the shared neighbors. The forth integer is used to distinguish those pairs whose former three integers are identical but have different atomic configurations. When the local configurations are described by the HA bond-type index method, it is well known that the 1551, 1541 and 1431 bond types are the characteristic bond types of typical liquids and amorphous states^7^,^37^, that 1441 and 1661 (6:8) are the characteristic bond types of a bcc crystal, and that 1421 and 1422 are the characteristic bond types of fcc (12:0) and hcp (6:6) crystals.

### Cluster type index method (CTIM)

**Figure 9:**
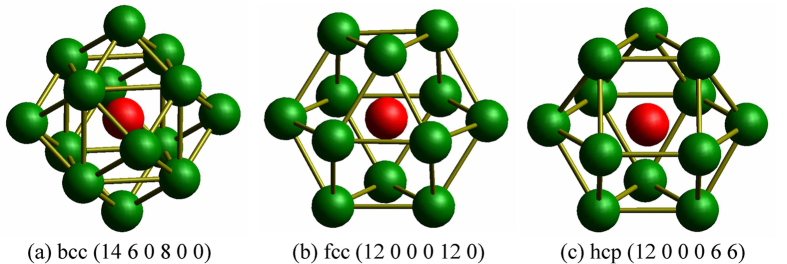
3D graphs of the three typical basic clusters of (**a**) bcc, (**b**) fcc and (**c**) hcp (for clarity, bonds between the center atoms and the near neighbors are omitted).

However, the HA bond-type indices are incapable of discerning clusters formed by an atom with its nearest neighbors, especially in the case of large clusters formed by different basic clusters. Therefore, based on the work of Qi and Wang^38^, Liu *et al*.^19^,^39^ proposed the cluster type index method (CTIM) to overcome this difficulty. In CTIM, a basic cluster is defined as the structure composed of a core atom and its surrounding neighbor atoms, and a set of four integers is adopted to describe the basic cluster. The first integer represents the total number of the neighbors near to the center atom, and the following three integers indicate the numbers of the 1441, 1551 and 1661 HA bond types, by which the surrounding atoms are connected with the center atom. With the CTIM, many amorphous and crystalline structures can be described exactly and clearly. However, some basic crystal clusters, such as fcc and hcp basic clusters composed of 1421 and 1422 bond types, cannot be described by CTIM. Therefore, we have added two integers (the fifth and sixth integers) to CTIM to represent the 1421 and 1422 bond types, respectively, in a basic cluster. This modified method is referred to as CTIM-2 21,22. With CTIM-2, the basic clusters of bcc, hcp and fcc can be exactly expressed as (14 6 0 8 0 0), (12 0 0 0 6 6) and (12 0 0 0 12 0), respectively, as shown in Fig. 9(a–c). Accordingly, the center atoms of bcc, hcp and fcc basic clusters can be identified as the bcc, hcp and fcc atoms, respectively. Based on CTIM-2, larger clusters composed of different types of basic clusters and bonded central atoms can be clearly described. Accordingly, in this paper, the large clusters, which consist of the bonded fcc and/or hcp atoms, are identified as crystalline nuclei (because only fcc and hcp type crystal structures appeared in the zinc system during the crystallization).

### Tracing method

The evolutions of nuclei during nucleation and growth were analyzed using the tracing method to identify the critical nucleus. The tracing method was first proposed by Honeycutt and Anderson^40^ and was then developed by Hou *et al*.^20^,^23^,^41^. This method first selects a cluster of interest at *t* = *t*_0_ and then traces backward in time to find its predecessor cluster with the most atoms in common with the cluster of interest at *t* = *t*_0_ − Δ*t*. The common atoms are regarded as inherited atoms. Then, the details of the evolution of the crystallization process can be traced, and the heredity [=N_0_ (number of the inherited atom)/N_1_ (size of the traced cluster)] can be defined. Heredity is a good measurement of the stability of the traced cluster. Additionally, the formation and evolution of the nuclei can be readily detected using the tracing method.

## Additional Information

**How to cite this article**: Zhou, L.-l. *et al*. Crystallization characteristics in supercooled liquid zinc during isothermal relaxation: A molecular dynamics simulation study. *Sci. Rep.* **6**, 31653; doi: 10.1038/srep31653 (2016).
